# Editorial: The immunosuppressive microenvironment in pediatric cancers: applications and considerations in immunotherapy

**DOI:** 10.3389/fonc.2025.1582467

**Published:** 2025-03-18

**Authors:** Jiaxiong Tan, Fei Gao, Ling Xu

**Affiliations:** ^1^ National Clinical Research Center for Cancer, Tianjin Medical University Cancer Institute and Hospital, Tianjin, China; ^2^ Brown Center for Immunotherapy, Indiana University School of Medicine, Indianapolis, IN, United States; ^3^ Institute of Hematology, School of Medicine, Jinan University, Guangzhou, China

**Keywords:** cancers, immunosuppressive microenvironment, immunotherapy, neuroblastoma, acute myeloid leukemia, acute lymphoblastic leukemia

Immunotherapy has truly transformed the landscape of cancer treatment, leading to remarkable improvements in survival rates across a broad spectrum of adult cancers. However, results to date in the pediatric cohort have been disappointing, largely due to differences in tumor biology, including high heterogeneity in the tumor immune microenvironment and generally low tumor mutation burden. These microenvironments, populated by immunosuppressive cells such as regulatory T cells (Tregs), myeloid-derived suppressor cells (MDSCs), and tumor-associated macrophages (TAMs), dampen the immune response against tumors and a number of complex molecular pathways drive tumor progression and drug resistance (1–3). Understanding and targeting these immunosuppressive mechanisms is crucial for the development of effective immunotherapeutic strategies in pediatric cancers.

In “*Mechanisms and Molecular Characterization of Relapsed/Refractory Neuroblastomas*”, Chen and Wei provided a thorough and insightful review of the complex molecular pathways that drive relapsed and refractory neuroblastomas, a pediatric cancer with a particularly grim prognosis. The authors highlighted key molecular alterations, such as MYCN amplification, ALK mutations, TERT promoter mutations, p53 pathway inactivation, and chromosomal instability, that contribute to the immunosuppressive microenvironment and therapeutic resistance. These molecular changes not only drive tumor progression but also create an environment that is hostile to immune cells. The review highlights the potential of precision medicine approaches targeting these molecular mechanisms to improve treatment outcomes, suggesting that combining immunotherapy with targeted therapies could enhance efficacy. Notably, MYCN amplification occurs in one-third of high-risk neuroblastomas (4). However, MYCN is a transcription factor and Intrinsically Disordered Proteins (IDPs), which makes it difficult to target MYCN directly (4, 5).

Building on these insights, He and Wang, in their article “*Targeting the Ubiquitin-Proteasome System: A Novel Therapeutic Strategy for Neuroblastoma*”, delved into the role of the ubiquitin-proteasome system (UPS) in neuroblastoma. They highlighted its potential as a therapeutic target. Their study presents an alternative and indirect strategy to modulate multiple challenging targets, such as MYCN and p53. The authors discussed how the UPS regulates protein stability, localization, and function—critical factors for tumor cell survival and proliferation. They also examined the impact of the UPS on neuroblastoma cell proliferation, apoptosis, and migration, along with the potential of targeting deubiquitination enzymes (DUBs) to enhance therapeutic efficacy. Preclinical studies on UPS inhibitors, such as bortezomib, have shown promising results in neuroblastoma models, suggesting that UPS-targeted therapies could be combined with immunotherapy to improve treatment outcomes.

In *“Development of a prognostic model incorporating a cuproptosis-related signature and CNN3 as a predictor in childhood acute myelocytic leukemia*”, Cao et al. investigated the role of cuproptosis-related genes (CRGs) in childhood acute myeloid leukemia (cAML) and developed a prognostic model based on these genes. The study identified 12 CRGs associated with patient outcomes, demonstrating significant differences in immune cell infiltration and drug sensitivity between high-risk and low-risk groups. In addition, the cuproptosis-related genes, calponin 3 (CNN3) and leucine-rich repeat-containing G-protein-coupled receptor 4 (LGR4, also called GPR48) were identified as modulators of cAML progression, and they were shown to be associated with immune cell infiltration, making them potential therapeutic targets. This research highlights the importance of understanding the molecular mechanisms of cell death in cancer cells, providing new insights into personalized treatment strategies for cAML. Additionally, the study underscores the importance of considering age-specific differences in the immune landscape when developing immunotherapies for pediatric cancers. In the study “*TRIM8 as a predictor for prognosis in childhood acute lymphoblastic leukemia based on a signature of neutrophil extracellular traps*”, Tin et al. explored the prognostic value of neutrophil extracellular traps (NETs) in childhood acute lymphoblastic leukemia (cALL). The study identified TRIM8 as a key gene associated with NETs and demonstrates its role in leukemia cell proliferation and prognosis. The findings suggest that TRIM8 knockdown improves outcomes in ALL models. The study also provided insight into the relationship between NET-related genes and immune cell communication, suggesting that targeting NETs may enhance the efficacy of immunotherapy. This research underscores the importance of understanding the complex interactions between cancer cells and the immune system, particularly in the context of pediatric cancers, where the immunosuppressive microenvironment poses significant challenges to treatment.

This Research Topic covers advances in immunology, genomics, and bioinformatics in common childhood tumors such as neuroblastoma, cAML, and cALL. Of the seven articles submitted, four were accepted for publication, comprising two reviews and two original research articles. Taken together, these contributions offer novel insights into the immunosuppressive microenvironment and immunotherapy in pediatric tumors, aiming to provide a foundation for advancing the application and therapeutic efficacy of immunotherapy in this vulnerable patient population. It is our hope that the content of this Research Topic will not only inspire readers but also draw increased attention from researchers to the unique challenges and opportunities presented by pediatric tumors, ultimately leading to more valuable research advances in this field.
